# Estimates of Maize Plant Density from UAV RGB Images Using Faster-RCNN Detection Model: Impact of the Spatial Resolution

**DOI:** 10.34133/2021/9824843

**Published:** 2021-08-21

**Authors:** K. Velumani, R. Lopez-Lozano, S. Madec, W. Guo, J. Gillet, A. Comar, F. Baret

**Affiliations:** ^1^Hiphen SAS, 120 Rue Jean Dausset, Agroparc, Bâtiment Technicité, 84140 Avignon, France; ^2^INRAE, UMR EMMAH, UMT CAPTE, 228 Route de l'Aérodrome, Domaine Saint Paul-Site Agroparc CS 40509, 84914 Avignon Cedex 9, France; ^3^Arvalis, 228, Route de l'Aérodrome-CS 40509, 84914 Avignon Cedex 9, France; ^4^International Field Phenomics Research Laboratory, Institute for Sustainable Agro-Ecosystem Services, Graduate School of Agricultural and Life Sciences, The University of Tokyo, Tokyo, Japan

## Abstract

Early-stage plant density is an essential trait that determines the fate of a genotype under given environmental conditions and management practices. The use of RGB images taken from UAVs may replace the traditional visual counting in fields with improved throughput, accuracy, and access to plant localization. However, high-resolution images are required to detect the small plants present at the early stages. This study explores the impact of image ground sampling distance (GSD) on the performances of maize plant detection at three-to-five leaves stage using Faster-RCNN object detection algorithm. Data collected at high resolution (GSD ≈ 0.3 cm) over six contrasted sites were used for model training. Two additional sites with images acquired both at high and low (GSD ≈ 0.6 cm) resolutions were used to evaluate the model performances. Results show that Faster-RCNN achieved very good plant detection and counting (rRMSE = 0.08) performances when native high-resolution images are used both for training and validation. Similarly, good performances were observed (rRMSE = 0.11) when the model is trained over synthetic low-resolution images obtained by downsampling the native training high-resolution images and applied to the synthetic low-resolution validation images. Conversely, poor performances are obtained when the model is trained on a given spatial resolution and applied to another spatial resolution. Training on a mix of high- and low-resolution images allows to get very good performances on the native high-resolution (rRMSE = 0.06) and synthetic low-resolution (rRMSE = 0.10) images. However, very low performances are still observed over the native low-resolution images (rRMSE = 0.48), mainly due to the poor quality of the native low-resolution images. Finally, an advanced super resolution method based on GAN (generative adversarial network) that introduces additional textural information derived from the native high-resolution images was applied to the native low-resolution validation images. Results show some significant improvement (rRMSE = 0.22) compared to bicubic upsampling approach, while still far below the performances achieved over the native high-resolution images.

## 1. Introduction

Plant density at emergence is an essential trait for crops since it is the first yield component that determines the fate of a genotype under given environmental conditions and management practices [1–5]. Competition between plants within the canopy depends on the sowing pattern and its understanding requires reliable observations of the plant localization and density [6–9]. An accurate estimation of actual plant density is also necessary to evaluate the seed vigor by linking the emergence rate to the environmental factors [10–13].

Maize plant density is measured by visual counting in the field. However, this method is labor intensive, time consuming, and prone to sampling errors. Several higher throughput methods based on optical imagery have been developed in the last twenty years. This was permitted by the technological advances with the increasing availability of small, light, and affordable high spatial resolution cameras and autonomous vehicles. Unmanned ground vehicles (UGV) provide access to detailed phenotypic traits [14–16] while being generally expensive and associated with throughputs of the order of few hundreds of microplots per hour. Conversely, unmanned aerial vehicles (UAV) are very affordable with higher acquisition throughput than UGVs. When carrying very high-resolution cameras, they can access potentially several traits [17, 18] including plant density [19, 20].

Image interpretation methods used to estimate plant density can be classified into three main categories. The first one is based on machine learning where the plant density measured over a small set of sampling area is related to other canopy level descriptors including vegetation indices derived from RGB and multispectral data [21–23]. However, this type of method may lead to significant errors due to the lack of representativeness of the training dataset as well as the effect of possible confounding factors including changes in background properties or plant architecture under genetic control. The second category of methods is based on standard computer vision techniques, where the image is first binarized to identify the green objects that are then classified into plants according to the geometrical features defined by the operator (e.g. [24, 25]). The last category of methods widely used now is based on deep learning algorithms for automatic object detection [26–28].

The main advantage of deep learning methods is their ability to automatically extract low-level features from the images to identify the targeted objects. Although deep learning methods appear very promising, their generalization capacity is determined by the volume and diversity of the training dataset [29]. While large collections of images can now be easily acquired, labeling the images used to train the deep models represents a significant effort that is the main limiting factor to build very large training datasets. Few international initiatives have been proposed to share massive labeled datasets that will contribute to maximize the performances of deep learning models [30–34], with however questions regarding the consistency of the acquisition conditions and particularly the ground sampling distance (GSD).

The use of UAV images for plant detection at early stages introduces important requirements on image resolution, as deep learning algorithms are sensitive to object scales with the identification of small objects being very challenging [35, 36]. For a given camera, low-altitude flights are therefore preferred to get the desired GSD. However, low-altitude flights decrease the acquisition throughput because of a reduced camera swath forcing to complete more tracks to cover the same experiment and require additionally to slow down the flying speed to reduce motion blur. An optimal altitude should therefore be selected to compromise between the acquisition throughput and the image GSD. Previous studies reporting early-stage maize plant detection from UAVs from deep learning methods did not addressed specifically this important scaling issue [20, 26, 27]. One way to address this scaling issue is to transform the low-resolution images into higher resolution ones using super resolution techniques. Dai et al. [37] have demonstrated the efficiency of super resolution techniques to enhance segmentation and edge detection. Later, Fromm et al. [38] and Magoulianitis et al. [39] showed improvements in object detection performances when using the super resolution methods. The more advanced super resolution techniques use deep convolutional networks trained over paired high- and low-resolution images [40–42]. Since the construction of a real-world paired high- and low-resolution dataset is a complicated task, the high-resolution images are often degraded using a bicubic kernel or less frequently using Gaussian noise to constitute the low-resolution images [43]. However, more recent studies have shown the drawbacks of the bicubic downsampling approaches as it smoothens sensor noise and other compression artifacts, thus failing to generalize while applied to real world images [41]. More recent studies propose the use of unsupervised domain translation techniques to generate realistic paired datasets for training the super resolution networks [44].

We propose here to explore the impact of image GSD on the performances of maize plant detection at stages from three to five leaves using deep learning methods. More specifically, three specific objectives are targeted: (1) to assess the accuracy and robustness of deep learning algorithms for detecting maize plants with high-resolution images used both, in the training and validation datasets; (2) to study the ability of these algorithms to generalize in the resolution domain, i.e. when applied to images with higher and lower resolution compared to the training dataset; and (3) to evaluate the efficiency of data augmentation and preparation techniques in the resolution domain to improve the detection performances. Special emphasis was put here on assessing the contribution of two contrasting methods to upsample low-resolution images: a simple bicubic upsampling algorithm and a more advanced super resolution model based on GAN (generative adversarial network) that introduces additional textural information. Data collected over several sites across France with UAV flights completed at several altitudes providing a range of GSDs were used.

## 2. Materials and Methods

### 2.1. Study Sites

**Figure 1:**
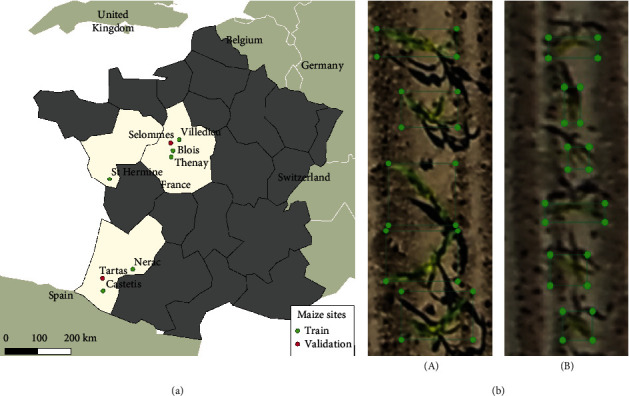
Location of the study sites with example extracts of the maize microplots acquired from UAV. (a) A map displaying the location of the eight maize phenotyping platforms located in the west of France used in this study. (b) An illustration of the bounding boxes drawn around the maize plants. The examples shown are from the Tartas site (GSD = 0.27 cm) (A) and Tartas site (GSD = 0.63 cm) (B).

This study was conducted over 8 sites corresponding to different field phenotyping platforms distributed across the west of France and sampled from 2016 to 2019 (Figure 1). The list of sites and their geographic coordinates are given in Table 1. Each platform included different maize microplots with size 20 to 40 square meters. Depending on the experimental design of the platform, the microplots were sown with two to seven rows of maize of different cultivars and row spacing varying from 30 to 110 cm. The sowing dates were always between mid-April and mid-May.

### 2.2. Data Acquisition and Processing

UAV flights were carried out on the eight sites approximately one month after the sowing date, between mid-May and mid-June (Table 1). Maize plants were in between three and five leaf stage, ensuring that there is almost no overlap among individual plants from near nadir viewing. The microplots were weeded and consisted of only maize plants. Three different RGB cameras were used for the data acquisition: Sony Alpha (ILCE-6000) with a focal length of 30 mm, DJI X7 (FC6540) with focal lengths of 24 mm and 30 mm, and the default camera with DJI Mavic 2 pro (L1D-20c) with a focal length of 10.26 mm mounted on AltiGator Mikrokopter (Belgium) and DJI Mavic 2 pro (China). To georeference the images, ground control points (GCPs) were evenly distributed around the sites and their geographic coordinates were registered using a Real-Time Kinematic GPS.

The flights were conducted at an altitude above the ground ranging between 15 and 22 meters, providing a ground sampling distance (GSD) between 0.27 and 0.35 cm (Table 1). For the Tartas and Selommes sites, an additional flight was done at a higher altitude on the same day providing a GSD between 0.63 and 0.66 cm.

The flights were planned with a lateral and front overlap of 60/80% between individual images. Each dataset was processed using PhotoScan Professional (Agisoft LLC, Russia) to align the overlapping images by automatic tie point matching, optimize the aligned camera positions, and finally georeference the results using the GCPs. The steps followed are similar to the data processing detailed by Madec et al. [15]. Once orthorectified, the multiple instances of the microplot present in the overlapping images were extracted using Phenoscript, software developed within the CAPTE research unit. Phenoscript allows to select, among the individual images available for each microplots, those with full coverage of the microplot, minimum blur, and view direction closer to the nadir one. Only these images were used in this study.

### 2.3. Manual Labeling of Individual Plants

**Figure 2:**
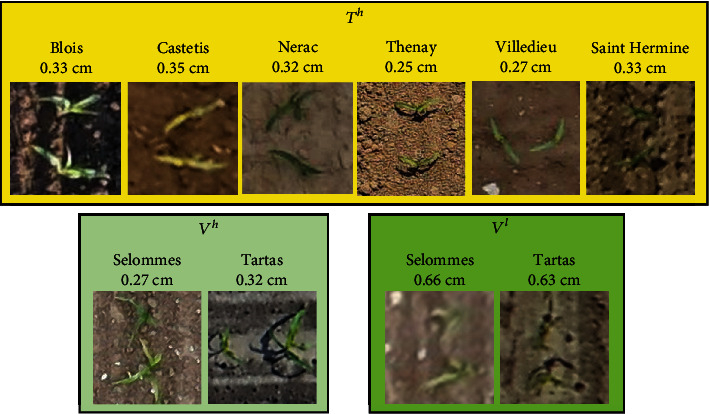
Examples of maize plants extracted from the in the eight sites used in this study. The image titles indicate the location of the sites. *T*^*h*^, *V*^*h*^, and *V*^*l*^ are the training high-resolution dataset, validation high-resolution dataset, and the validation low-resolution dataset, respectively.

From each site, the microplots were labeled with an offline tool, LabelImg [45]: bounding boxes around each maize plant were interactively drawn (Figure 1(b)) and saved in the Pascal VOC format as XML files. The available sites (Table 1) were divided into three groups: (1) the first group (*T*^*h*^) composed of six sites was used to train the plant detection models. It includes a total of 202 microplots corresponding to 19,841 plants. (2) The second group (*V*^*h*^) corresponding to the Tartas and Selommes with low-altitude flights was used to evaluate the model performance at high resolution. It includes a total of 36 microplots corresponding to 3256 plants. (3) The third group (*V*^*l*^) corresponds to the high-altitude flights in Tartas and Selommes was used to evaluate the model performance at low resolution. It includes a total of 36 microplots corresponding to 3256 plants. An example of images extracted from the three groups is shown in Figure 2.

### 2.4. The Faster-RCNN Object Detection Model

Faster-RCNN [46], a convolutional neural network designed for object detection, was selected to identify maize plants in the image. Besides its wide popularity outside the plant phenotyping community, Faster-RCNN has also been proved to be suitable for various plant and plant-organ detection tasks [47–49]. We used the implementation of Faster-RCNN in the open-source MMDetection Toolbox [50], written in PyTorch, with pretrained weights on ImageNet. The Faster-RCNN model with a ResNet50 backbone was trained for 12 epochs with a batch size of 2. The weights were optimized using an SGD optimizer (stochastic gradient descent) with a learning rate of 0.02. For the model training, ten patches of 512 × 512 pixels were randomly extracted from each microplot in the training sites. Standard data augmentation strategies such as rotate, flip, scale, brightness/contrast, and jpeg compression were applied.

### 2.5. Experimental Plan

To evaluate the effect of the resolution on the reliability of maize plant detection, we compared Faster-RCNN performances over training and validation datasets made of images of high (GSD ≈ 0.30 cm) and low (GSD ≈ 0.60 cm) resolution. Three training datasets built from *T*^*h*^ (Table 1) were considered: (1) the original *T*^*h*^ dataset with around 0.32 cm GSD; (2) a dataset, *T*_*gm*_^*h*⟶*l*^ where the images from *T*^*h*^ were downsampled to 0.64 cm GSD using a Gaussian filter and motion blur that mimics the actual low-resolution imagery acquired at higher altitude as described later (Section 2.6.1); and (3) a dataset, where the original *T*^*h*^ high-resolution dataset was merged with its low-resolution transform, *T*_*gm*_^*h*⟶*l*^. This *T*^*h*^ + *T*_*gm*_^*h*⟶*l*^ is expected to provide robustness of the model towards changes in GSD. Note that we did not investigate the training with the native low-resolution images because the labeling of low-resolution images is often difficult because plants are not easy to identify visually and to draw accurately the corresponding bounding box. Further, only two flights were available at the high altitudes (Table 1) that were reserved for the validation. A specific model was trained over each of the three training datasets considered (Table 2) and then evaluated over independent high- and low-resolution validation datasets.

We considered three validation datasets for the high-resolution images: (1) the native high-resolution validation dataset, *V*^*h*^ acquired at low altitude with GSD around 0.30 cm (Table 1); (2) a synthetic high-resolution dataset of GSD around 0.30 cm obtained by upsampling the native low-resolution dataset, acquired at high altitude, using a bicubic interpolation algorithm as described in Section 2.6.2, and it will be called *V*_*bc*_^*l*⟶*h*^; and (3) a synthetic high-resolution dataset, *V*_*sr*_^*l*⟶*h*^, obtained by applying a super resolution algorithm (see Section 2.6.3) to the native low-resolution dataset *V*^*l*^ and resulting in images with a GSD around 0.30 cm. Finally two low-resolution datasets will be also considered: (1) the native low-resolution validation dataset, *V*^*l*^ (Table 1), with a GSD around 0.60 cm and (2) a synthetic low-resolution dataset, *V*_*gm*_^*h*⟶*l*^, obtained by applying a Gaussian filter to downsample (see Section 2.6.1) the original high-resolution dataset, *V*^*h*^, and get a GSD around 0.60 cm.

### 2.6. Methods for Image Up- and Downsampling

#### 2.6.1. Gaussian Filter Downsampling

**Figure 3:**
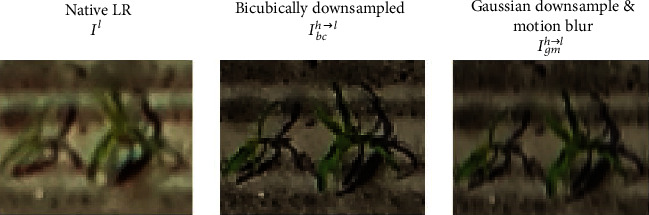
Visual comparison of the extract of the same plant from the Tartas site between different versions of low resolution. Native low resolution, synthetic low resolution from bicubic downsampling, and synthetic low resolution from Gaussian downsampling (sigma = 0.63, window = 9) followed by a motion blur (kernel size = 3 and angle = 45).

To create the synthetic low-resolution datasets *T*_*gm*_^*h*⟶*l*^ and *V*_*gm*_^*h*⟶*l*^, a Gaussian filter with a sigma = 0.63 and a window size = 9 followed by a motion blur with a kernel size = 3 and angle = 45 were applied to downsample the native high-resolution datasets *T*^*h*^ and *V*^*h*^ by a factor of 2. This solution was preferred to the commonly used bicubic downsampling method because it provides low-resolution images more similar to the native low-resolution UAV images (Figure 3). This was confirmed by comparing the image variance over the Selommes and Tartas sites where both native high- and low-resolution images were available: the variance of the *V*_*gm*_^*h*⟶*l*^ was closer to that of *V*^*l*^ whereas the bicubic downsampled dataset had a larger variance corresponding to sharper images. This is consistent with [38, 51] who used the same method to realistically downsample high-resolution images.

#### 2.6.2. Bicubic Upsampling

The bicubic interpolation algorithm was used to generate *V*_*bc*_^*l*⟶*h*^ by upsampling the native low-resolution UAV images, *V*^*l*^. The bicubic interpolation available within Pillow, the Python Imaging Library [52], was used to resample the images.

#### 2.6.3. Super Resolution Images Derived from Cycle-ESRGAN

The super resolution (SR) is an advanced technique that artificially enhances the textural information while upsampling images. We used a SR model inspired from [53]. It is a two-stage network composed of a CycleGAN network that generates synthetic paired data and a ESRGAN network capable of image upsampling. The CycleGAN [54] performs unsupervised domain mapping between the native low-resolution and bicubic downsampled low-resolution domains. Thus, for any given input image, CycleGAN is trained to add realistic image noise typical of low-resolution images. The ESRGAN-type super resolution network [42] upsamples by a factor of two low-resolution images.

**Figure 4:**
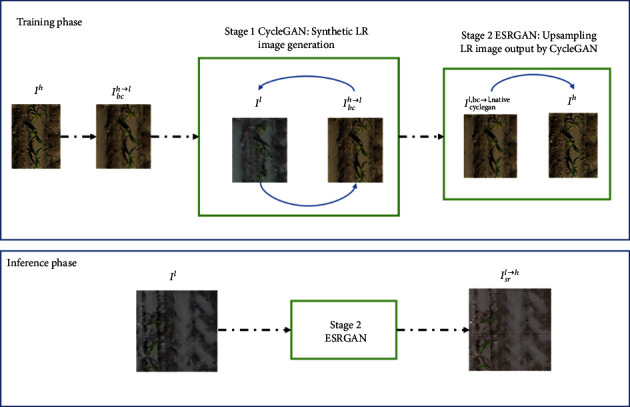
An illustration of the super resolution pipeline. *I*^*h*^ and *I*^*l*^ are the native high- and low-resolution images, respectively. *I*_*bc*_^*h*⟶*l*^ and *I*_cyclegan_^*h*⟶*l*^ are the synthetic low-resolution images prepared from the native high-resolution images by bicubic downsampling and by the CycleGAN network, respectively. *I*_*sr*_^*l*⟶*h*^ is the synthetic high-resolution image generated by the super resolution network.

During the training phase, the CycleGAN stage of the network was trained to generate “real-world” noise from an unpaired dataset of native low-resolution and bicubically downsampled images. The second stage of the network consisting of the ESRGAN was then trained using a paired dataset of native high-resolution image and a “realistic” low-resolution image generated by the CycleGAN (Figure 4). The CycleGAN stage of the network was initially trained for a few epochs following which the two stages (CycleGAN+ESRGAN) were trained together simultaneously. It should be noted that during inference, only the ESRGAN stage of the network would be activated. Hence, for a given input image, the ESRGAN network would upsample the input by a factor of 2. The training parameters and losses reported by Han et al. [53] were used for the model training. The model weights were initialized over the Div2k dataset [55] and finetuned on the UAV dataset detailed below. The Cycle-ESRGAN network was implemented using Keras [56] deep learning library in Python. The codes will be made available on Github at the following link: https://github.com/kaaviyave/Cycle-ESRGAN.

A dedicated training dataset for the super resolution network was prepared using UAV imagery belonging to the following two domains:

- Native high-resolution domain: 2234 microplot extractions from four sites with an average GSD of less than 0.33 cm. Some of the sites belonging to the *T*^*h*^dataset was used as a part of the training
- Native low-resolution domain: 1713 microplot extractions from three sites with an average GSD of 0.46 cm per site

None of the validation sites (*V*^*l*^ and *V*^*h*^ in Table 1) were used in the training of the super resolution model. The synthetic downsampled dataset used to train the CycleGAN was prepared by bicubic downsampling the native high-resolution domain by a factor of 2. The images were split into patches of size 256 × 256 pixels for the high-resolution domain and into 128 × 128 pixels for the low-resolution domain.

### 2.7. Evaluation Metrics

In this study, the average precision (AP), root mean squared error (RMSE), and accuracy will be utilized for the evaluation of the Faster-RCNN models for the purpose of maize plant detection and counting.

AP is a frequently used metric for the evaluation of object detection models and can be considered the area under the precision-recall curve.

(1) $$\begin{array}{c} \text{Precision}=\frac{\text{TP}}{\text{TP}+\text{FP}},\\ \text{Recall}=\frac{\text{TP}}{\text{TP}+\text{FN}}, \end{array}$$

where TP is the number of true positive, FP is the number of false positive, and the FN is the number of false negative. For the calculation of AP, a predicted bounding box is considered: true positive (TP) if its intersection area over union area (IoU) with the corresponding labeled bounding box is larger than a given threshold. Depending on the objective of the study, different variations exist in the AP metric calculation and the choice of IOU threshold used to qualify a predicted bounding box as TP. After considering several IoU threshold values, we decided to use an IoU threshold of 0.25 to compute AP. This will be later justified. The Python COCO API was used for the calculation of the AP metric [57].

Accuracy evaluates the model's performance by calculating the ratio of correctly identified plants to all the predictions made by the model. A predicted bounding box is considered true positive if it has a confidence score of more than 0.5 and an IoU threshold of 0.25. Accuracy is then calculated as

(2) $$\begin{array}{c} \text{Ac}=\frac{\text{TP}}{\text{TP}+\text{FP}+\text{FN}}. \end{array}$$

The relative root mean square error (rRMSE) between the number of labeled and detected plants across all images belonging to the same dataset:

(3) $$\begin{array}{c} \text{rRMSE}=\frac{\sqrt{\sum_{i}^{n}{\left(P_{o,i}-P_{p,i}\right)}^{2}/n}}{\bar{P_{o,1}}}, \end{array}$$

where *P*_*o*,*i*_ is the number of plants labeled on image and *P*_*p*,*i*_ is the number of images predicted by the CNN (confidence score > 0.5 and an IoU > 0.25) and $\bar{P_{o,l}}$ is the average number of labeled plants per image.

## 3. Results and Discussion

### 3.1. Faster-RCNN Detects Plants with High Accuracy at High Spatial Resolution

**Figure 5:**
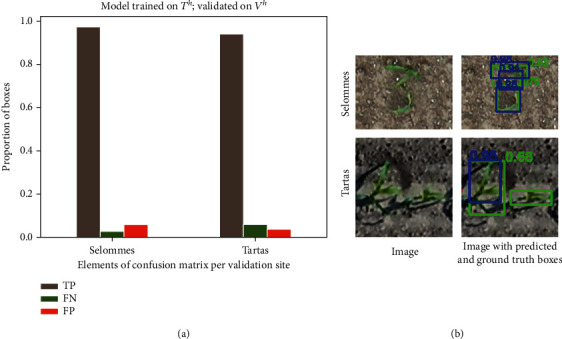
Results of the model trained on the native HR dataset, *T*^*h*^, and applied to the HR validation dataset, *V*^*h*^. (a) Elements of the confusion matrix—true positives (TP), false negatives (FN), and false positives (FP) for the Selommes and Tartas sites. (b) An example of false positive and false negative observed in the two validation sites. The ground truth bounding boxes are shown in green, and the predicted bounding boxes are shown in blue. The green text indicates the IoU of the predicted bounding box with the ground truth, and the blue text indicates the confidence score of the predictions.

Very good performances (rRMSE = 0.08; Ac = 0.88, AP = 0.95) are achieved when the model is trained over the high-resolution images (*T*^*h*^) and applied on high-resolution images taken on independent sites (*V*^*h*^). The good performances are explained by the high rate of true positives (Figure 5(a)). However, the detector performs slightly differently on the two sites used for the validation: in Selommes, an overdetection (false positives, FP) is observed for a small number of plants, when the detector splits a plant into two different objects (Figure 5(b)). Conversely, in the Tartas site, some underdetection (false negatives, FN) is observed, with a small number of undetected plants (Figure 5).

**Figure 6:**
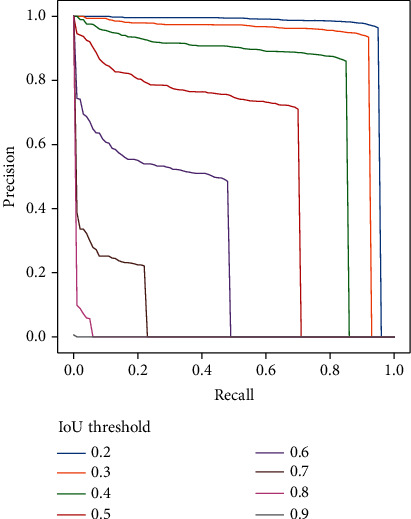
Precision-recall curve at different IoU thresholds for the plant detection model trained and applied with high-resolution images (*T*^*h*^, *V*^*h*^).

A detailed analysis of the precision-recall curves for the configuration [*T*^*h*^, *V*^*h*^] at different IoU (Figure 6) shows a drastic degradation of the detector performances when the IoU is higher than 0.3. This indicates that the model is not accurate when determining the exact dimensions of maize plants. This is partly explained by the difficulty of separating the green from the ground in the shadowed parts of the images. As a consequence, some shaded leaves are excluded from the bounding boxes proposed by the detector and, conversely, some shadowed grounds are wrongly included in the bounding boxes proposed (Figure 5(b)). Further, when a single plant is split into two separate objects by the detector, the resulting bounding boxes are obviously smaller than the corresponding plant (Figure 5(b)). As a consequence, we proposed to use an IoU threshold of 0.25 to evaluate the model performance to better account for the smaller size of the detected bounding boxes. This contrasts from most object detection applications where an IoU threshold of 0.5 or 0.75 is commonly used to evaluate the performance of the methods [58, 59]. The observed degradation of the model performance for IoU above 0.3 indicates that the method presented provides less accurate localization than in other object detection studies, including both, real world objects and phenotyping applications [49, 60, 61]. An inaccurate estimation of plant dimensions is not critical for those applications assessing germination or emergence rates and uniformity, where plant density is the targeted phenotypic trait. If the focus is to additionally assess the plant size in early developmental stages as well, mask-based RCNN [62, 63] could be used instead. In contrast to algorithms trained on rectangular regions like Faster-RCNN, mask-based algorithms have the potentials to more efficiently manage the shadow projected on the ground by plants, limiting therefore the possible confusion between shaded leaves and ground during the training. However, generating mask annotations is time consuming, increasing the effort needed to generate a diverse training dataset.

These results provide slightly better performances as those reported by David et al. [20] with Ac ≈ 0.8 and rRMSE ≈ 0.1 when using the “out-domain” approach as the one used in this study, i.e. when the training and validation sites are completely independent. They used images with a spatial resolution around 0.3 cm as in our study. This is also consistent with the results of Karami et al. [26] who obtained an accuracy of 0.82 with a spatial resolution of around 1 cm. They used the anchor-free few shot leaning (FSL) method which identifies and localizes the maize plants by estimating the central position. They claim that their method is a little sensitive to object size and thus to the spatial resolution of the images. The accuracy increases up to 0.89 when introducing few images from the validation sites in the training dataset. Kitano et al. [27] proposed a two-step method: they first segment the images using a CNN-based method and then count the segmented objects. They report an average rRMSE of 0.24 over a test dataset where many factors including image resolution vary (ranging from GSD ≈ 0.3 cm to 0.56 cm). They report that their method is sensitive to the size and density of the objects. In the following, we will further investigate the dependency of the performances to image resolution.

### 3.2. The Faster-RCNN Model Is Sensitive to Image Resolution and Apparent Plant Size

**Figure 7:**
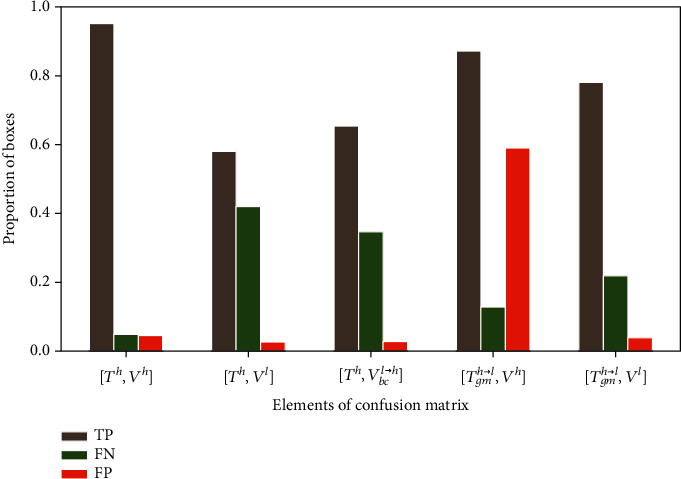
Results of maize plant detection when trained and evaluated across different resolution domains. *T*^*h*^: native high-resolution training dataset; *T*_*gm*_^*h*⟶*l*^: low-resolution training dataset by downsampling *T*^*h*^ using Gaussian motion blur; *V*^*h*^: native high-resolution validation dataset; *V*^*l*^: native low-resolution validation dataset; *V*_*bc*_^*l*⟶*h*^: high-resolution dataset by upsampling *V*^*l*^ using bicubic interpolation.

The performances of the model were evaluated when it is trained and validated over images with different resolution. When Faster-RCNN is trained on the high-resolution domain (*T*^*h*^) and applied to a dataset with low resolution (*V*^*l*^), both AP and Ac decrease almost by 30% (Table 3) compared to the results where the model is trained and applied over high-resolution images. The rate of true positive drops because of the drastic increase of false negatives indicating a high rate of misdetection (Figure 7, [*T*^*h*^, *V*^*l*^]). This degradation of the detection performances impacts highly the rRMSE that increases up to 0.48. This indicates that the model is sensitive to the resolution of the images. We further investigated if this was linked to the apparent size of the plants and therefore upsampled the validation low-resolution images with a bicubic interpolation method (*V*_*bc*_^*l*⟶*h*^) to get plants with the same size as in the native high-resolution images (*V*^*h*^). Results show that Ac increases from 0.54 to 0.63 and AP from 0.64 to 0.77. However, because of the high imbalance between FN and FP (Figure 7, [*T*^*h*^, *V*_*bc*_^*l*⟶*h*^]), the counting performances remains poor with rRMSE = 0.49.

**Figure 8:**
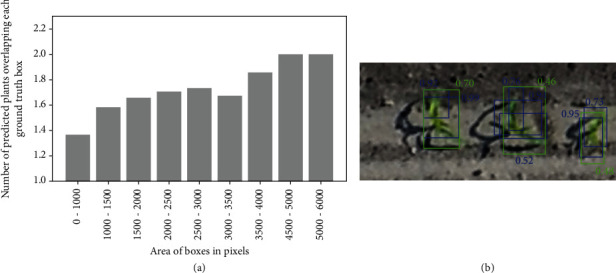
Effects of the hyperspecialization of Faster-RCNN trained with synthetic low-resolution images (*T*_*gm*_^*h*⟶*l*^) and applied to a high-resolution dataset (*V*^*l*^). (a) Relationship between the size of the ground truth bounding boxes and the average number of predicted bounding boxes intersecting with them. (b) Example of overdetection of maize plants due to different object size. The ground truth bounding boxes are shown in green, and the predicted boxes are shown in blue. The green text indicates the IoU of the predicted boxes with the ground truth, and the blue text indicates the confidence score of the predictions.

When the model is trained over simulated low-resolution images (*T*_*gm*_^*h*⟶*l*^), the detection and counting performances evaluated on high-resolution images (*V*^*h*^) also degrades drastically (Table 3). The rate of true positive is relatively high, but the rate of false positive increases drastically (Figure 7(*T*_*gm*_^*h*⟶*l*^, *V*^*h*^)). We observe that the average number of predicted bounding boxes overlapping each labeled box increases linearly with its size (Figure 8). For example, the model identifies on average two plants inside plants larger than 4000 pixels. The imbalance between FN and FP explains the very poor counting performances with rRMSE = 0.52 (Table 3). This result confirms the importance to keep consistent the resolution and plant size between the training and the application datasets since Faster-RCNN tends to identify objects that have a similar size to the objects used during the training.

We thus evaluated whether data augmentation may improve the performances on the low-resolution images (*V*^*l*^): the Faster-RCNN model trained on the simulated low-resolution images (*T*_*gm*_^*h*⟶*l*^) shows improved detection performances as compared to the training over the native high-resolution images (Table 3) with a decrease of the rRMSE down to 0.29 (Table 3). When this model trained with synthetic low-resolution images (*T*_*gm*_^*h*⟶*l*^) is applied to a dataset downscaled to a similar resolution (*V*_*gm*_^*h*⟶*l*^), the performances improve dramatically with Ac increasing from 0.56 to 0.89 and AP from 0.71 to 0.90 while the rRMSE drops to 0.10. However, when this model trained with synthetic low-resolution images (*T*_*gm*_^*h*⟶*l*^) is applied to the native low-resolution images (*V*^*l*^), moderate detection performances are observed which degrades the counting estimates with rRMSE = 0.29 (Table 3).

**Figure 9:**
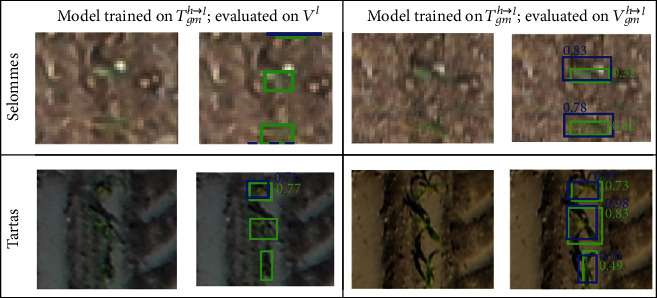
An example showing the same plants extracted from the exact same locations in two versions of the validation dataset: native LR (*V*^*l*^) and the synthetic LR obtained from Gaussian downsampling (*V*_*gm*_^*h*⟶*l*^). The first and third columns show the raw images while the second and fourth columns show the detector predictions. The ground truth bounding boxes are shown in green, and the predicted bounding boxes are shown in blue. The green text indicates the IoU of the predicted box with the ground truth, and the blue text indicates the confidence score of the predictions.

The performances of the model trained over the synthetic low-resolution images (*T*_*gm*_^*h*⟶*l*^) are quite different when evaluated over the native images (*V*^*l*^) or the synthetic ones (*V*_*gm*_^*h*⟶*l*^) with the latter yielding results almost comparable to the high-resolution configurations with AP = 0.90 (Table 3). This indicates that the low-resolution synthetic images contain enough information to detect accurately the maize plants. Conversely, the native low-resolution image, *V*^*l*^, has probably lost part of the textural information. In addition, the model trained on the synthetic low-resolution images is not able to extract the remaining pertinent plant descriptors from the native low-resolution images. We can observe that the native low-resolution images contain less details as compared to the synthetic ones (Figure 9): some plants are almost not visible in the *V*^*l*^ images, as the textural information vanishes and even the color of maize leaves cannot be clearly distinguished from the soil background. This explains why the model was not able to detect the plants, even when it is trained with the synthetic low-resolution images (*T*_*gm*_^*h*⟶*l*^).

Contrary to vectors that operate at an almost constant height like ground vehicles [16, 64–66] or fixed cameras [67–70], camera settings (aperture, focus and integration time) in UAVs need to be adapted to the flight conditions, especially flight altitude, to maximize image quality. Further, the jpg recording format of the images may also significantly impact image quality. Recording the images in raw format would thus improve the detection capability at the expense of increased data volume and sometimes image acquisition frequency.

### 3.3. Data Augmentation Makes the Model More Resistant to Changes in Image Resolution

We finally investigated whether mixing high- and low-resolution images in the training dataset would make the model more resistant to changes in the image resolution. Results show that merging native high-resolution with synthetic low-resolution images (*T*^*h*^ + *T*_*gm*_^*h*⟶*l*^) provides (Table 4) performances similar to those observed when the model is trained only over high (*T*^*h*^) or synthetic low (*T*_*gm*_^*h*⟶*l*^) and validated on the same resolution (*V*^*h*^ or *V*_*gm*_^*h*⟶*l*^) (Table 3). This proves that data augmentation could be a very efficient way to deal with images having different resolutions. Further, this model trained on augmented data (*T*^*h*^ + *T*_*gm*_^*h*⟶*l*^) (Table 4) surprisingly beats the performances of the model trained only on the high-resolution images (*T*^*h*^) as displayed in Table 3. This is probably a side effect of the increase of the size of the training dataset (Table 2). Nevertheless, when validating on the native low-resolution images (*V*^*l*^) (Table 4), the performances are relatively poor as compared to the model trained only on the synthetic low-resolution images (*T*_*gm*_^*h*⟶*l*^). This is explained by the lower quality of the native low-resolution images as already described in the previous section.

### 3.4. Upsampling with the Super Resolution Method Improves the Performances of Plant Detection on the Native Low-Resolution Images

If the training is difficult with the native low-resolution images because plants are visually difficult to identify and label, the training should be done over low-resolution images derived from the high-resolution images using a more realistic upsampling method than the standard bicubic interpolation one. Alternatively, the training could be done using the high-resolution images and the low-resolution dataset may be upsampled to a synthetic high-resolution domain using bicubic interpolation or super resolution techniques.

**Figure 10:**
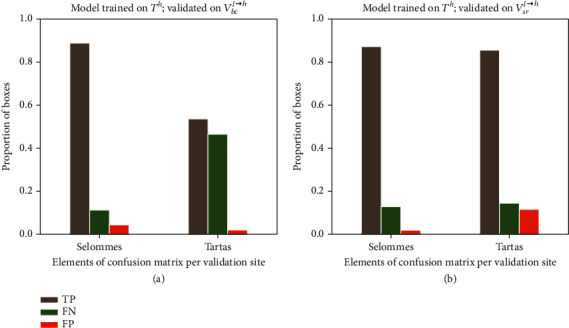
A comparison between the performance of the Faster-RCNN model trained on the HR dataset, *T*^*h*^, and applied to the synthetic high-resolution datasets. (a) Model trained on *T*^*h*^ and evaluated on the synthetic HR dataset *V*_*bc*_^*l*⟶*h*^, from the bicubic upsampling. (b) Model trained on *T*^*h*^ applied to the synthetic HR dataset **V**_**sr**_^**l**⟶**h**^, from the super resolution technique.

Results show that the super resolution technique improved plant detection very significantly as compared to the native low-resolution (*V*^*l*^) and bicubic upsampled (*V*_*bc*_^*l*⟶*h*^) images (Table 5). This impacts positively the counting performances while not reaching the performances obtained with the high-resolution images (*V*^*h*^). The super resolution reduces drastically the underdetection of maize plants particularly on the Tartas site (Figure 10), where as mentioned in Section 3.2, these native low-resolution images have lower textural information and green fraction per plant.

**Figure 11:**
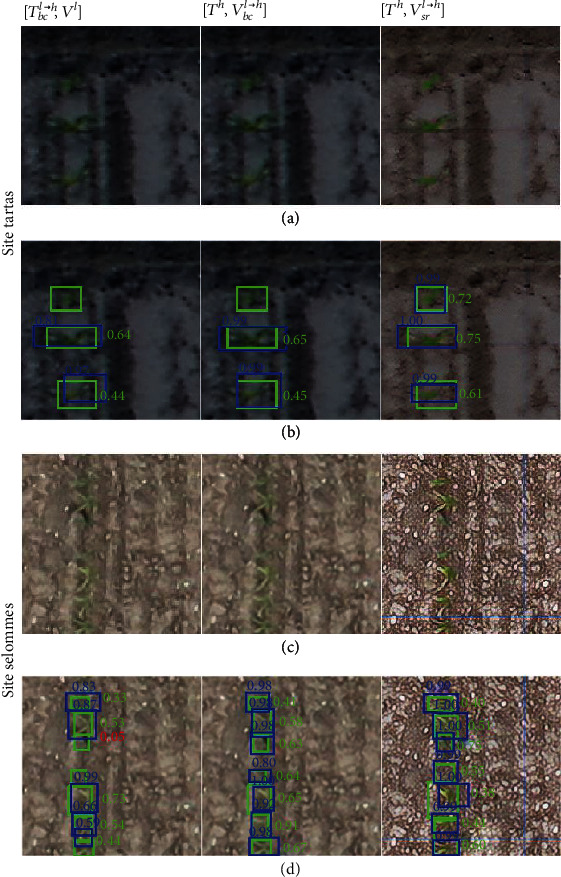
Illustration of the performance of the Faster-RCNN model on the synthetic high-resolution and native low-resolution datasets. (a, c) Images belonging to three datasets: native low resolution *V*^*l*^, bicubically upsampled *V*_*bc*_^*l*⟶*h*^, and finally the upsampling by super resolution technique *V*_*sr*_^*l*⟶*h*^. (b, d) The results predicted by the model trained on *T*_*gm*_^*h*⟶*l*^ applied to *V*^*l*^ (first column) and the model trained on *T*^*h*^ applied to the synthetic high-resolution datasets *V*_*bc*_^*l*⟶*h*^ and *V*_*sr*_^*l*⟶*h*^ (second and third columns). The ground truth bounding boxes are shown in green, and the predicted bounding boxes are shown in blue. The green text indicates the IoU of the predicted bounding box with the ground truth, and the blue text indicates the confidence score of the predictions.

The super resolution approach enhances the features used to identify maize plants, with colors and edges more pronounced than in the corresponding native LR images (Figure 11). Maize plants are visually easier to recognize in the superresolved images as compared to both the native low-resolution and the bicubically upsampled images.

**Figure 12:**
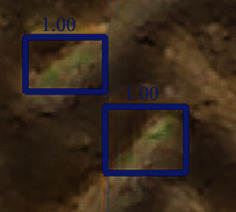
An example where the super resolution approach adds undesired artifacts in the image leading to false positives during detection. The SR model adds a few green pixels to the robot tracks on the soil which have “leaf-like” texture. This is wrongly detected as leaf by the Faster-RCNN model. The ground truth boxes are shown in green, and the predicted boxes are shown in blue. The green text indicates the IoU of the predicted box with the ground truth, and the blue text indicates the confidence score of the predictions.

Nevertheless, although easier to interpret, the images generated by super resolution do not appear natural with some exaggerated textural features of the soil background (Figures 11(c) and 11(d)). In few cases, super resolution images show new features—e.g., coloring some pixels in green—in leaf-shaped shadows or tractor tracks in the background (Figure 12) leading to an increase in the proportion of false positives in certain microplots of the Tartas site (Figure 10(b)). Training the super resolution model with a larger dataset might help the generator network to limit those artifacts. Alternatively, some studies [39, 71, 72] have proposed to integrate the training of the super resolution model with the training of the Faster-RCNN. The use of a combined detection loss would provide additional information on the location of the plants, thus forcing the super resolution network to differentiate between plants and background while upsampling the images.

In terms of computation speed, the super resolution network takes approximately 20 s to upsample a low-resolution image of 878 × 250 pixels. The detection of the maize plants using the Faster-RCNN model takes approximately 1.5 s for a low-resolution image of 878 × 250 pixels with *x* objects whereas it takes roughly 4 s for its high-resolution counterpart of size 623 × 2337 pixels. Thus, the super resolution and prediction on a high-resolution image is almost 10 times computationally more expensive than predicting directly on a low-resolution image. All the computations were clocked using a graphical processing unit NVIDIA GEFORCE 1080i with a memory of 12 GB.

## 4. Conclusion

We evaluated the performances of automatic maize plant detection from UAV images using deep learning methods. Our results show that the Faster-RCNN model achieved very good plant detection and counting (rRMSE = 0.08) performances when high-resolution images (GSD ≈ 0.3 cm) are used both for training and validation. However, when this model is applied to the low-resolution images acquired at higher altitudes, the detection and counting performances degrade drastically with rRMSE = 0.48. We demonstrated that this was mostly due to the hyperspecialization of Faster-RCNN that is expecting plants of similar size as in the training dataset. The sensitivity of the detection method to the object size is a critical issue for plant phenotyping applications, where datasets can be generated from different platforms (UAVs, ground vehicles, portable imaging systems, etc.) each one of them providing images within at a specific ground resolution. Concurrently, it would be optimal to share labeled images to get a wide training dataset. Data augmentation techniques where high- and low-resolution images populate the training dataset were proved to be efficient and provide performances similar to the ones achieved when the model is trained and validated over the same image resolution. However, the native low-resolution images acquired from the UAV have significant low quality that prevents accurate plant detection. In some cases, the images are difficult to visually interpret which poses a problem both for their labeling and for the detector to localize plants due to the lack of pertinent information. These low-quality images were characterized by a loss of image texture that could come from camera intrinsic performances, inadequate settings, and the jpg recording format. It is thus recommended to pay great attention to the camera choice, settings, and recording format when the UAV is flying at altitudes that provide resolution coarser than 0.3 cm for maize plant counting.

In the future studies, it would be worth evaluating the model performances over datasets acquired over a range of flying altitudes, to identify an optimal flying altitude and data quality for plant detection. In our study, it was demonstrated that the quality of the synthetic low-resolution dataset was highly dependent on the low-altitude images used for resampling. Thus, evaluating the model performances at a range of GSDs using additional synthetic low-resolution datasets from Gaussian motion blur resampling would not be representative of the real-world acquisition conditions. It would hence be more pertinent to develop specific metrics allowing to evaluate the richness of textural information contained in the images, rather than using GSD as the main criteria to evaluate image quality.

Finally, we evaluated a super resolution Cycle-ESRGAN-based method to partially overcome the problem of suboptimal image quality. The super resolution method significantly improved the results on the native low-resolution dataset compared to the classic bicubic upsampling strategies. However, the performances when applied to the native low-resolution images were moderate and far poorer than those obtained with the native high-resolution images with simulated superresolved images showing sometimes artifacts. A future direction to reduce the artifacts of such super resolution algorithms can be to integrate the GAN training along with the training of the plant detection network. Another direction would be to introduce some labeled low-resolution images in the training dataset to possibly integrate their features in model. It would also be worth evaluating the performance of more recent object detection networks for plant counting tasks. For instance, one-stage object detection networks such as Yolo-v5 [73]/Yolo-v4 [74] or RetinaNet [75] that outperform Faster-RCNN, would increase the data processing throughput. However, their ability to handle small-scale objects and sensitivity to data quality needs to be studied.

## Figures and Tables

**Table 1:** The dataset used for the training and validation of the object detection models are listed here.

Dataset	#	Site name	Latitude (°)	Longitude (°)	Acquisition date	Camera	Flight altitude (m)	Focal length (mm)	GSD (cm)	No. microplots labeled	No. plants labeled	Average plant size (pixels)
*T* ^*h*^	1	Castetis	43.46	-0.71	06-06-2019	FC6540	26	24	0.35	20	2239	939
	2	St. Hermine	46.54	-1.06	23-05-2019	FC6540	26	35	0.33∗	20	2674	806
	3	Nerac	44.16	0.3	01-06-2017	ILCE-6000	25	30	0.32	44	3338	2253
	4	Thenay	47.38	1.28	18-05-2018	ILCE-6000	22	30	0.25	72	7454	1505
	5	Villedieu	47.88	1.53	28-06-2016	n/a	n/a	n/a	0.27	26	2390	2159
	6	Blois	47.56	1.32	18-05-2018	ILCE-6000	25	30	0.33	20	1746	1419

*V* ^*h*^	7	Tartas	43.80	-0.79	08-06-2019	FC6540	20	24	0.32	22	2151	1336
	8	Selommes	47.76	1.19	17-05-2019	L1D-20c	16.2	10.26	0.27	14	1105	891

*V* ^*l*^	9	Tartas	43.80	-0.79	08-06-2019	FC6540	40	24	0.63	24	2151	437
	10	Selommes	47.76	1.19	17-05-2019	L1D-20c	30	10.26	0.66	14	1105	156

*T*^*h*^ is the training high-resolution dataset, *V*^*h*^ is the validation high-resolution dataset, and *V*^*l*^ is the validation low-resolution dataset. ^∗^For this site, the microplot extracts were resampled to GSD = 0.25 cm before annotating.

**Table 2:** Description of the training and validation datasets.

	Dataset name	No. microplots	No. plants	Comment
Training	*T* ^*h*^	202	19,841	Native high-resolution training dataset
	*T*_*gm*_^*h*⟶*l*^	202	19,841	Downsampling *T*^*h*^ with Gaussian filter and motion blur
	*T*^*h*^+*T*_*gm*_^*h*⟶*l*^	404	39,682	Merging *T*^*h*^ and *T*^*h*⟶*l*^

Validation	*V* ^*h*^	36	3256	Native high-resolution validation dataset
	*V*_*bc*_^*l*⟶*h*^	36	3256	Upsampling *V*^*l*^with bicubic algorithm
	*V*_*sr*_^*l*⟶*h*^	36	3256	Upsampling *V*^*l*^with Cycle-ESRGAN super resolution
	*V* ^*l*^	36	3256	Native low-resolution validation dataset
	*V*_*gm*_^*h*⟶*l*^	36	3256	Downsampling *V*^*h*^ with gaussian filter and motion blur

**Table 3:** Comparison of the performance of the Faster-RCNN models trained and validated over datasets with different resolutions.

		Validation					
		High resolution (*V*^*h*^)			Low resolution (*V*^*l*^)		
		rRMSE	Ac	AP	rRMSE	Ac	AP
Training	*T* ^*h*^	0.08	0.88	0.95	0.48	0.54	0.64
	*T*_*gm*_^*h*⟶*l*^	0.52	0.56	0.71	0.29	0.76	0.81

**Table 4:** Comparison of the performance of the Faster-RCNN models trained over the augmented data (*T*^*h*^ + *T*_*gm*_^*h*⟶*l*^) and validated over datasets with different resolutions.

	rRMSE	Ac	AP
*V* ^*h*^	0.06	0.91	0.96
*V*_*gm*_^*h*⟶*l*^	0.11	0.92	0.91
*V* ^*l*^	0.48	0.64	0.72

**Table 5:** Comparison of the performance of the Faster-RCNN models trained over high-resolution images and applied to the native low-resolution images (*V*^*l*^), the synthetic high-resolution images that is upsampled/transformed using either bicubic (*V*_*bc*_^*l*⟶*h*^) or super resolution (*V*_*sr*_^*l*⟶*h*^) techniques.

	rRMSE	Ac	AP
*V* ^*l*^	0.58	0.54	0.64
*V*_*bc*_^*l*⟶*h*^	0.43	0.63	0.77
*V*_*sr*_^*l*⟶*h*^	0.22	0.80	0.85

## Data Availability

The UAV data (microplot extractions with bounding boxes) used to support the findings of this study are available from the corresponding authors upon request.
